# Combining Minimally Invasive Direct Coronary Artery Bypass Grafting with Transapical Aortic Valve Implantation—The Next Level Heart Team Approach

**DOI:** 10.3390/jcm12216890

**Published:** 2023-11-01

**Authors:** Jules Miazza, Ion Vasiloi, Luca Koechlin, Brigitta Gahl, David Santer, Denis Berdajs, Thomas Nestelberger, Christoph Kaiser, Friedrich Eckstein, Oliver Reuthebuch

**Affiliations:** 1 Department of Cardiac Surgery, University Hospital Basel, 4031 Basel, Switzerland; jules.miazza@usb.ch (J.M.); david.santer@usb.ch (D.S.);; 2 Department of Cardiology, University Hospital Basel, University of Basel, 4031 Basel, Switzerland

**Keywords:** TAVR, MIDCAB, intervention, cardiac surgery

## Abstract

We present the results of a combined approach for transapical aortic valve replacement and minimally invasive coronary artery bypass grafting (taTAVI-MIDCAB) in patients with combined aortic stenosis and coronary artery disease. Background: For patients presenting with aortic stenosis and coronary artery disease, a simultaneous procedure addressing both diseases is recommended to reduce operative risk. In high-risk patients with hostile femoral or coronary axis, taTAVI-MIDCAB can be an alternative minimally invasive approach, offering the benefits of left interior mammary artery to left anterior descending coronary artery (LIMA-LAD) grafting. Methods: From 2014 to 2022, 10 patients underwent taTAVI-MIDCAB for combined coronary and severe aortic stenosis in the hybrid operation theater at our institution. We assessed perioperative outcomes and follow-up outcomes. Results: The median age was 83 years (81 to 86). The procedure was successfully performed in all patients without conversion to sternotomy. The median length of hospital and intensive care unit stay was 9 days (7 to 16) and 2.5 days (1 to 5), respectively. The median flow over the coronary artery bypass was 31 (22 to 44) mL/min, with a pulsatility index (PI) of 2.4 (2.1 to 3.2). Mild paravalvular leak occurred in 2 patients (10%). There were no neurological events nor acute kidney injury. Pacemaker implantation was required in 1 patient (10%). Conclusions: Simultaneous surgical coronary revascularization and interventional valve implantation in the setting of a hostile femoral and coronary axis appears to be safe and beneficial.

## 1. Introduction

Since the first aortic valve replacement using a homograft in 1962 [1], aortic valve surgery has undergone remarkable development. The relentless innovation in this field has led to less invasive open-heart surgery, as well as interventional techniques. Originally conceptualized in the late 1980s [2], Transcatheter Aortic Valve Implantation (TAVI) rapidly gained popularity in the treatment of severe aortic stenosis (AS). Nowadays, it is a class IA recommendation for patients over 75 years of age, for high-risk patients, or for patients unsuitable for open-heart surgery according to the 2021 guidelines of the European Society of Cardiology (ESC) [3]. As of today, the vast majority of TAVI is usually implanted via a percutaneous transfemoral access. However, in cases where the femoral axis is prohibited, trans-subclavian, transcarotid, or transapical approaches are feasible and may be considered for patients not suited for surgery [3,4]. 

To complete the preoperative assessment, coronary angiography is recommended in patients undergoing TAVI. In the presence of coronary artery disease (CAD) with stenosis over 70%, peri-interventional myocardial revascularization should be performed in order to reduce procedural risk [3]. In parallel to the advent of interventional techniques in the treatment of severe AS, myocardial revascularization has also evolved toward less invasive approaches over recent decades, with the development of percutaneous coronary interventions (PCI), in addition to minimally invasive coronary artery bypass grafting (CABG) [5,6]. Despite the appeal for fully catheter-based therapies of coronary artery disease, CABG remains the treatment of choice in diabetes, reduced left-ventricular function, complex lesion anatomy, left main disease, and three-vessel disease [7]. Furthermore, minimally invasive strategies for coronary revascularization have been proposed. The minimally invasive direct coronary artery bypass grafting (MIDCAB) is performed via a left anterior minithoracotomy and allows for revascularization of lesions of the left anterior descending artery and the circumflex artery [8,9].

Consequently, for patients with combined AS and coronary artery disease, careful assessment of patient- and procedure-related factors should be conducted in order to provide optimal patient care. Interdisciplinary decision making should be strived for in the context of the heart team. In recent years, a novel approach to combined aortic and coronary artery disease composed of transapical TAVI and MIDCAB (taTAVI-MIDCAB) has been proposed and has proven to be safe and feasible [10,11,12]. In a retrospective study, TAVI-MIDCAB showed slightly poorer outcomes when compared with transfemoral TAVI and percutaneous coronary intervention [13]. However, this study was not primarily designed to analyze taTAVI-MIDCAB but consisted of a group of taTAVI with either off-pump coronary artery bypass grafting (OPCAB) or MIDCAB. Consequently, the generally scarce evidence and lack of randomized data do not allow us to draw definitive conclusions for the role of taTAVI-MIDCAB in the treatment of patients with combined aortic and coronary artery disease. 

In this retrospective single-center study, we aim to analyze in-hospital and follow-up outcomes of patients undergoing taTAVI-MIDCAB from 2014 to 2022 at our institution.

## 2. Materials and Methods

A retrospective, single-center analysis was performed at the University Hospital of Basel, Switzerland. We included ten patients who underwent taTAVI-MIDCAB for concomitant severe AS and CAD from May 2014 to September 2022. Perioperative patients’ characteristics were collected routinely using the institutional prospectively maintained quality management software (Dendrite Clinical Systems, V1.7), and were regularly checked for completeness and consistency. Follow-up data were obtained from patients medical records. Perioperative and postoperative outcomes were defined according to valve academic research consortium 3 criteria [14]. Data were presented as mean and standard deviation, median and interquartile range, or number and %. Statistical analyses were performed by a biostatistician (BG).

### 2.1. Patient Selection

Patients with severe AS and significant CAD involving the left anterior descending artery treated with taTAVI-MIDCAB were included. The diagnosis of severe AS was made according to the 2021 ESC/EACTS Guidelines for the Management of valvular heart disease [3]. Significant CAD was defined as stenosis over 70% in the coronary angiogram. In each case, the patients diagnosis, comorbidities, and suitability for either surgical or interventional therapy were discussed in the context of an interdisciplinary heart team. In our cohort, the anatomy of patient’s coronary, femoral, and iliac arteries played a key role in patient selection. In the era of interventional cardiac therapy, high-risk patients with severe AS and concomitant CAD are—up to a certain degree—treated in a fully percutaneous manner, combining transcatheter aortic valve implantation and PCI. However, there is a small proportion of patients with unsuitable vascular anatomy for a transfemoral TAVI. Furthermore, some patients are not amenable for PCI due to a hostile coronary anatomy (e.g., chronic total occlusion, aneurysmatic dilatation, or multiple, heavily calcified stenoses). In certain clinical scenarios, patients combine complex femoral and coronary anatomy. In our cohort, those factors predominantly drove the decision to perform taTAVI-MIDCAB. One patient suffered from inflammatory skin affections in the groin. Justification for taTAVI is depicted in Table 1.

### 2.2. Surgical Technique 

**Figure 1:**
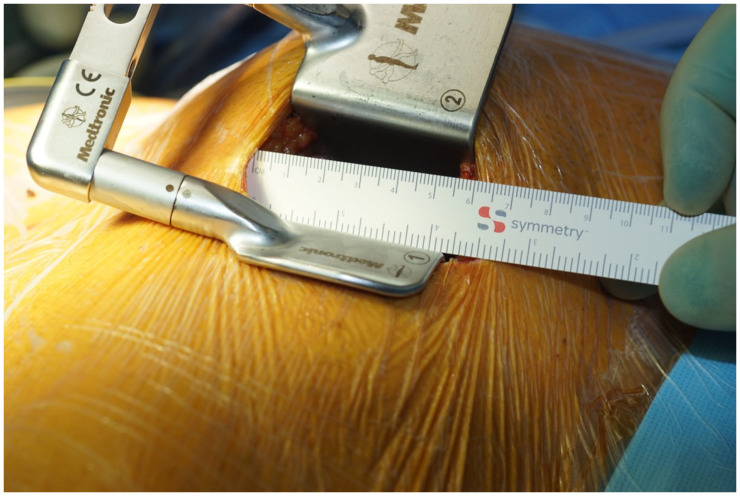
Left-sided anterior minithoracotomy.

**Figure 2:**
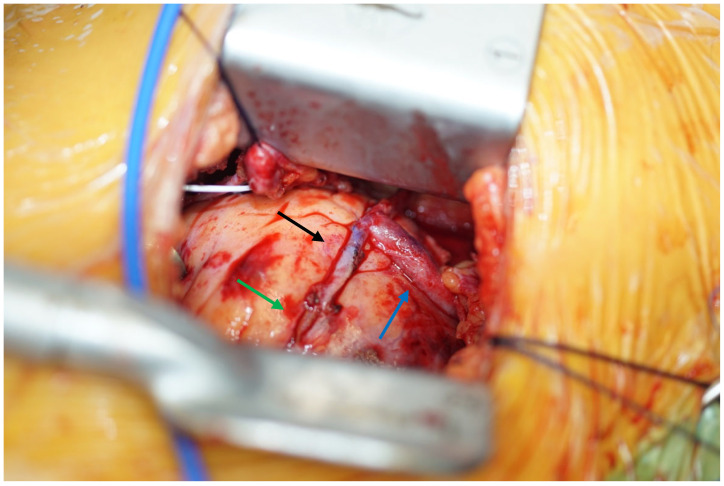
Exemplary intraoperative situs: end-to-side anastomosis between the left IMA (black arrow) and the left anterior descending artery (green arrow), as well as T-graft anastomosis using a great saphenous vein (blue arrow) segment.

All procedures are performed in the hybrid operation theater. First, the heart apex is marked using transthoracic echocardiography. The surgical access is performed via a left-sided anterior minithoracotomy (Figure 1). Generally, the thorax is entered via the 4th or 5th intercostal space and the left internal mammary artery (IMA) is dissected in skeletonized fashion using the ThoraTrak retractor (Medtronic, Inc., Minneapolis, MN, USA) (Figure 1). If needed, a segment of the great saphenous vein can be simultaneously endoscopically harvested. After completion of the left IMA take-down, the end-to-side anastomosis between the left IMA and the left anterior descending artery (LAD) is performed using an 8-0 prolene running suture (Figure 2). 

After completion of the graft, run-off is controlled with transit time flow measurement (TTFM) [15]. Fluoroscopy is installed, the femoral artery is punctured for the mere guide wire to be inserted, and a pig-tail catheter is placed in the aortic root for angiographic visualization. Felt-pledgeted purse-string sutures are then placed around the apex and consequently punctured to allow for placement of the taTAVI delivery system. The valve is then positioned and deployed under fluoroscopic and echocardiographic monitoring.

## 3. Results

### 3.1. Preoperative Patients Characteristics 

From May 2014 to September 2022, ten patients underwent taTAVI-MIDCAB for concomitant severe aortic stenosis and coronary artery disease. The median (SD) age was 83 years [81 to 86] and 50% were female. All patients had significant stenosis of the left anterior descending artery. In one case, percutaneous coronary intervention (PCI) of the right coronary artery was performed preoperatively. The majority (70%, n = 7) of the patients had symptoms of dyspnea NYHA III to IV. The mean pressure gradient over the aortic valve was 39 mmHg [29 to 47] and the mean valve area was 0.55cm^2^ [0.38 to 0.83]. The median preoperative STS Mortality and EuroSCORE2 risk scores were 9.1% [7.3 to 10.3] and 9.7% [7.4 to 17.7], respectively. Preoperative patients’ characteristics are depicted in Table 2. 

### 3.2. Procedural Data

In all ten patients, a single left-sided minithoracotomy was used as surgical access. In 90% (n = 9), an Edward Sapien 3 valve (Edwards Lifesciences Corp., Irvine, CA, USA) was implanted. To perform MIDCAB, the left internal mammary artery (IMA) was used in 100% of the patients. In 90% (n = 10), one anastomosis was performed, while in one case, 2 anastomosis were performed using the left IMA and the great saphenous vein. The median operation time was 231 [225 to 251] minutes. In one case, postoperative PCI of ramus circumflexus was performed. Other stenosis were treated conservatively, according to the recommendation of the heart team. Procedural data are depicted in Table 3. 

### 3.3. Postoperative Outcomes

The procedure was performed successfully in 100%. There was no periprocedural cerebral complication, as well as no myocardial infarction and no bleeding at the site of thoracotomy. However, bleeding complications occurred in two patients (20%) comprising one case of epistaxis without the need for blood transfusion (Type 1 bleeding complication according to the VARC3 classification [14]) and one case of hematoma leading to revision after great saphenous vein harvesting (Type 3 bleeding complication according to the VARC3 classification [14]). In one case, postoperative permanent pacemaker implantation was required due to 3rd grade atrioventricular block grade. In-hospital mortality was 0%. The mean pressure gradient over the newly implanted valve was 8.6mmHg [8.0 to 13.0]. The length of intensive care unit (ICU) and hospital stay was 2.5 days [1.0 to 5.0] and 9.0 days [7.0 to 16], respectively. Postoperative outcomes are depicted in Table 4.

### 3.4. Follow-Up Data

Clinical follow-up was available for all patients. The median follow-up time was 332.5 [203.25 to 406] days. Mortality at follow-up was 20% (n = 2) and occurred at 3.8 and 4.2 years. There was no case of myocardial infarction or cerebral ischemic event. Echocardiographic follow-up was available in 64% (n = 7) after 267 [131 to 376 days]. The median left ventricular ejection fraction was 52.5% [46.25 to 58]. The mean pressure gradient over the newly implanted prosthesis was 8.6 mmHg [8 to 13] and there was one case of paravalvular leak (10%). Note that in one case of a patient with immediate postoperative paravalvular leak, no follow-up echocardiography was available at the time of the analysis. Follow-up data are depicted in Table 5.

## 4. Discussion

In this retrospective, observational, single-center study, we provide some of the first real-world data on a minimal invasive approach for the treatment of combined severe AS and CAD using transapical aortic valve replacement and minimally invasive coronary artery bypass grafting (taTAVI-MIDCAB). Specifically, we focused on patients in which the anatomy of coronary and femoral arteries presented complex lesions, thus making a fully percutaneous approach extremely unadvisable (Table 1). In our analysis, this technique showed to be safe and feasible in all of the patients. We report no in-hospital mortality, no perioperative myocardial infarction, and no perioperative stroke. After a mean follow-up time of 332.5 [203.5 to 406] days, mortality was 20% (n = 2), with no stroke and no reintervention linked to the index operation.

The combination of transapical aortic valve implantation and minimally invasive coronary artery bypass grafting for patients suffering from severe aortic stenosis and coronary artery disease has been proposed in previous studies [11,13]. When compared to surgical aortic valve replacement and coronary artery bypass grafting via sternotomy or transfemoral aortic valve implantation and percutaneous coronary intervention, this approach bore higher in-hospital mortality and 12-months posthospital mortality [13]. However, as Baumbach et al. [13] rightfully mention, their study lacked randomization, resulting in a heterogenous patient population between the groups reflecting real-world data on the use of this technique. Furthermore, in the study by Baumbach et al., no difference was made between patients treated with minimally invasive coronary artery bypass grafting vs. off-pump coronary artery bypass grafting.

In our cohort, we investigated the perioperative outcomes of patients treated only with taTAVI-MIDCAB. In all of the cases, the interdisciplinary heart team opted for taTAVI-MIDCAB due to the complex anatomy of patients’ femoral arteries, coronary arteries, or both. Indeed, this cohort is composed of high-risk patients with a mean EuroSCORE and STS-Score of 11% [7.4 to 18] and 8.3% [6.2 to 10], respectively. While technically feasible, the highly predicted perioperative risk of this patient cohort made it prohibitive to perform open-heart surgery. Furthermore, the complex vascular anatomy precluded for a fully percutaneous approach, which is why taTAVI-MIDCAB was proposed by the heart team.

In all of the cases, the procedure was performed successfully. First, MIDCAB was performed with a mean flow over the bypass of 31 [22 to 44] mL/min. Thereafter, taTAVI was successfully implanted in all patients via the same access. This high success rate is encouraging considering the high perioperative risk of this patient cohort.

The postoperative outcomes showed no myocardial infarction or stroke and only two (20%) cases of minor bleeding according to the VARC-3 criteria [14]. There was one case of new pacemaker implantation due to atrioventricular block grade III, which is on par with the available literature describing a rate of new pacemaker implantation ranging from 9 to 26% after transfemoral TAVI [16] and 6.2 to 8.5% [17,18] after taTAVI. However, it is important to mention that the small number of patients in our cohort preclude to draw definite conclusions on the rate of postoperative pacemaker implantation after taTAVI-MIDCAB.

Follow-up was available for 100% (n = 10) of the patients in our cohort. After a mean follow-up time of 332.5 [203.25 to 406 days], mortality was 20% (n = 2). No patient had to undergo reintervention linked to the index operation. In patients with available echocardiographic follow-up, the mean pressure gradient was 8.6 [8 to 13] mmHg, and one (14%) case of mild paravalvular leak was diagnosed. The results are encouraging and show that taTAVI-MIDCAB might represent at least a viable mid-term option for high risk patients combining coronary artery disease and severe AS with complex coronary and femoral arteries anatomy.

In the era of highly specialized medicine with evolving techniques and the increasing number of comorbidities of cardiac surgery patients [19,20,21], this analysis shows the importance of interdisciplinary teamwork to provide the best possible patient care. Indeed, cases such as those presented in this report are highly complex in nature and require direct collaboration between anesthesiologists, cardiologists, and cardiac surgeons, not only in the conference room but also in the hybrid operating theater.

### Limitations

Despite these encouraging results, this study has several limitations. First, this single-center observational analysis lacks randomization and comparison to other therapeutical approaches and focuses on high-risk patients with pronounced coronary and femoral arteries pathologies. Despite this methodological aspect, this study provides real-world data on a patient cohort that is difficult to integrate in a prospective, randomized analysis. Second, the small number of patients included does not allow us to draw definitive conclusions regarding the perioperative outcomes. Finally, even though follow-up was obtained for 100% of the patients, the mean follow-up time was less than a year. Consequently, further studies with longer follow-up times are needed to analyze the long-term results of taTAVI-MIDCAB.

## 5. Conclusions

In high-risk patients, particularly in those with hostile femoral and coronary axis, transapical aortic valve implantation combined with minimally invasive coronary artery grafting represents a safe and feasible therapy for patients not suited for open-heart surgery or fully percutaneous approaches. Perioperative outcomes show low rates of major adverse cardiovascular and cerebral events, despite high preoperative risk scores. This technique could represent a viable option in the era where cardiac surgery patients show an increasing number of comorbidities and should be the focus of further studies with larger cohorts to assess its role in today’s cardiology and cardiac surgery landscapes.

## Figures and Tables

**Table 1:** Reasons for performing taTAVI-MIDCAB for each patient.

Patient Number	Coronary Anatomy	Kinking of Femoral Vessels	Femoral Calcification	Other
1	X	X	X	
2			X	
3	X			
4	X		X	
5		X		
6	X		X	
7	X			X
8	X		X	
9	X	X		
10	X			

This table shows the reasons underlying the heart team’s decision to recommend taTAVI-MIDCAB rather than tfTAVI with PCI in our cohort. Other: seborrheic dermatitis of the groin.

**Table 2:** Preoperative patients’ characteristics.

Patient Characteristics	Total (N = 10)
Female sex	5 (50%)
Age (years)	83 [81 to 86]
Body Mass Index (kg/m^2^)	24 [23 to 31]
Hypertension	9 (90%)
Hypercholesterolemia	5 (50%)
Diabetes	5 (50%)
Smoking history	
Never smoked	6 (60%)
Ex-smoker	3 (30%)
Unknown	1 (10%)
Angina	
CCS 0	2 (20%)
CCS 1	6 (60%)
CCS 2	0 (0%)
CCS 3	1 (10%)
CCS 4	1 (10%)
Dyspnea	
NYHA 1	3 (30%)
NYHA 2	0 (0%)
NYHA 3	5 (50%)
NYHA 4	2 (20%)
Extracardiac arteriopathy	90 (90%)
Pulmonary artery systolic pressure	
Normal (<31 mmHg)	3 (30%)
Moderate (31–55 mmHg)	3 (30%)
Unknown	4 (40%)
Atrial fibrillation	5 (50%)
Previous cerebrovascular event	1 (10%)
Chronic pulmonary disease	1 (10%)
Left main CAD	5 (50%)
CAD with over 50% narrowing	10 (100%)
LAD	10 (100%)
Circumflex	3 (30%)
RCA	8 (80%)
Preoperative PCI	1 (10%)
Syntax II Score (%)	23 [13 to 34]
Creatinine clearance [mL/min]	42 [32 to 54]
Porcelain Aorta	4 (40%)
Aortic mean pressure gradient (mmHg)	39 [29 to 47]
Mean valve area (cm^2^)	0.55 [0.38 to 0.83]
Left Ventricular Ejection Fraction (%)	
<35	1 (10%)
35–50	7 (70%)
>50	2 (20%)
Aortic regurgitation more than moderate	1 (10%)
STS Score Mortality (%)	8.3 [6.2 to 10]
STS Morbidity and Mortality (%)	26 [22 to 37]
Logistic EuroSCORE (%)	26 [16 to 40]
EuroSCORE2 (%)	11 [7.4 to 18]

Data are presented as median and interquartile range or number and %. CCS, Canadian Cardiovascular Society; NYHA, New York Heart Association; CAD, Coronary Artery Disease; LAD, Left Anterior Descending; RCA, Right Coronary Artery; STS, Society of Thoracic Surgeons.

**Table 3:** Procedural data.

Procedural Data	Total (N = 10)
Access	
Left-sided minithoracotomy	10 (100%)
Distal coronary anastomosis	
1	9 (90%)
2	1 (9%)
Graft	
Left IMA	10 (100%)
Long/great SV	1 (9%)
Median flow over bypass (mL/min) Pulsatility Index	31 [22 to 44] 2.4 [2.1 to 3.2]
Prosthesis type	
JenaValve	1 (9%)
Edwards Sapien	9 (90%)
Prosthesis size (mm)	
23	2 (20%)
25	1 (10%)
26	4 (40%)
29	3 (30%)
Conversion to sternotomy	0 (0%)
Operative time (min)	231 [225 to 251]
Staged postoperative PCI	1 (10%)

Data are presented as median and interquartile range or number and %. IMA, Internal Mammary Artery; SV, Saphenous Vein.

**Table 4:** Postoperative outcomes.

Postoperative Outcomes	Total (N = 10)
Periprocedural MI	0 (0%)
Neurological complications	0 (0%)
Delirium	3 (30%)
Bleeding (VARC3)	
Type 1	1 (10%)
Type 2	0 (0%)
Type 3	1 (10%)
Type 4	0 (0%)
Rethoracotomy	0 (0%)
Acute kidney injury	0 (0%)
New pacemaker	1 (10%)
In-hospital mortality	0 (0%)
Mean pressure gradient (mmHg)	8.6 [8.0 to 13.0]
Paravalvular leak	
No	8 (80%)
Mild	2 (20%)
Moderate	0 (0%)
Severe	0 (0%)
New postoperative atrial fibrillation	3 (30%)
Length of ICU stay (days)	2.5 [1.0 to 5.0]
Length of hospital stay (days)	9.0 [7.0 to 16]

Data are presented as median and interquartile range or number and %. MI, Myocardial Infarction; VARC3, Valve Academic Research Consortium 3 [14]; ICU, Intensive Care Unit.

**Table 5:** Follow-up data.

Follow-Up Data	Total (N = 10)	Time to Event
Available follow-up	10 (100%)	
Follow-up time, days	332.5 [203.25 to 406]	
Mortality	2 (20%)	3.8 year, 4.2 year
Myocardial infarction	0 (0%)	
Stroke	0 (0%)	
Reintervention linked to index operation	0 (0%)	
Available echocardiographic follow-up	7 (64%)	
Mild paravalvular leak *	1 (14%)	
Echocardiographic follow-up time, days	267 [131 to 376]	
Left ventricular ejection fraction, %	52.5 [46.25 to 58]	
Mean aortic prosthesis gradient	8.6 [8 to 13]	

Data are presented as median and interquartile range or number and %. AF, Atrial Fibrillation; CAD, Coronary Artery Disease; COPD, Chronic Obstructive Pulmonary Disease; MUST-Score, Sternum malunion prediction scale; NYHA, New York Heart Association. * Calculated on the basis of n = 7 patients with available follow-up.

## Data Availability

Data available on request due to restrictions e.g., privacy or ethical.

## References

[1] Ross D.N. (1962). Homograft Replacement of the Aortic Valve. Lancet.

[2] Figulla H.R., Franz M., Lauten A. (2020). The History of Transcatheter Aortic Valve Implantation (TAVI)—A Personal View Over 25 Years of Development. Cardiovasc. Revasculariz. Med..

[3] Vahanian A., Beyersdorf F., Praz F., Milojevic M., Baldus S., Bauersachs J., Capodanno D., Conradi L., De Bonis M., De Paulis R. (2022). 2021 ESC/EACTS Guidelines for the Management of Valvular Heart Disease: Developed by the Task Force for the Management of Valvular Heart Disease of the European Society of Cardiology (ESC) and the European Association for Cardio-Thoracic Surgery (EACTS). Rev. Esp. Cardiol..

[4] Biasco L., Ferrari E., Pedrazzini G., Faletra F., Moccetti T., Petracca F., Moccetti M. (2018). Access Sites for TAVI: Patient Selection Criteria, Technical Aspects, and Outcomes. Front. Cardiovasc. Med..

[5] Mack M.J., Squiers J.J., Lytle B.W., DiMaio J.M., Mohr F.W. (2021). Myocardial Revascularization Surgery: JACC Historical Breakthroughs in Perspective. J. Am. Coll. Cardiol..

[6] Canfield J., Totary-Jain H. (2018). 40 Years of Percutaneous Coronary Intervention: History and Future Directions. J. Pers. Med..

[7] Neumann F.J., Sousa-Uva M., Ahlsson A., Alfonso F., Banning A.P., Benedetto U., Byrne R.A., Collet J.P., Falk V., Head S.J. (2019). 2018 ESC/EACTS Guidelines on Myocardial Revascularization. Eur. Heart J..

[8] Calafiore A.M., Di Giammarco G., Teodori G., Bosco G., D’Annunzio E., Barsotti A., Maddestra N., Paloscia L., Vitolla G., Sciarra A. (1996). Left Anterior Descending Coronary Artery Grafting via Left Anterior Small Thoracotomy without Cardiopulmonary Bypass. Ann. Thorac. Surg..

[9] Reuthebuch O., Stein A., Koechlin L., Gahl B., Berdajs D., Santer D., Eckstein F. (2022). Five-Year Survival of Patients Treated with Minimally Invasive Direct Coronary Artery Bypass (MIDCAB) Compared with the General Swiss Population. Thorac. Cardiovasc. Surg..

[10] Cheung A., Hon J.K.F., Ye J., Webb J. (2010). Combined Off-Pump Transapical Transcatheter Aortic Valve Implantation and Minimally Invasive Direct Coronary Artery Bypass. J. Card. Surg..

[11] Mayr B., Firschke C., Erlebach M., Bleiziffer S., Krane M., Joner M., Herold U., Nöbauer C., Lange R., Deutsch M.A. (2018). Transcatheter Aortic Valve Implantation and Off-Pump Coronary Artery Bypass Surgery: An Effective Hybrid Procedure in Selected Patients. Interact. Cardiovasc. Thorac. Surg..

[12] Langenaeken T., De Raeymaeker X., De Poortere A., Rega F., Oosterlinck W. (2017). Minimally Invasive Direct Coronary Artery Bypass and TAVI: Timing and Considerations in Octogenarians: A Case Report. Eur. J. Mol. Clin. Med..

[13] Baumbach H., Schairer E.R., Wachter K., Rustenbach C., Ahad S., Stan A., Hill S., Bramlage P., Franke U.F.W., Schäufele T. (2019). Transcatheter Aortic Valve Replacement- Management of Patients with Significant Coronary Artery Disease Undergoing Aortic Valve Interventions: Surgical Compared to Catheter-Based Approaches in Hybrid Procedures. BMC Cardiovasc. Disord..

[14] Généreux P., Piazza N., Alu M.C., Nazif T., Hahn R.T., Pibarot P., Bax J.J., Leipsic J.A., Blanke P., Blackstone E.H. (2021). Valve Academic Research Consortium 3: Updated Endpoint Definitions for Aortic Valve Clinical Research. J. Am. Coll. Cardiol..

[15] Gaudino M., Sandner S., Di Giammarco G., Di Franco A., Arai H., Asai T., Bakaeen F., Doenst T., Fremes S.E., Glineur D. (2021). The Use of Intraoperative Transit Time Flow Measurement for Coronary Artery Bypass Surgery: Systematic Review of the Evidence and Expert Opinion Statements. Circulation.

[16] Rück A., Saleh N., Glaser N. (2021). Outcomes Following Permanent Pacemaker Implantation After Transcatheter Aortic Valve Replacement: SWEDEHEART Observational Study. JACC Cardiovasc. Interv..

[17] D’Ancona G., Pasic M., Unbehaun A., Hetzer R. (2011). Permanent Pacemaker Implantation after Transapical Transcatheter Aortic Valve Implantation. Interact. Cardiovasc. Thorac. Surg..

[18] Ewe S.H., Delgado V., Ng A.C.T., Antoni M.L., Van Der Kley F., Marsan N.A., De Weger A., Tavilla G., Holman E.R., Schalij M.J. (2011). Outcomes after Transcatheter Aortic Valve Implantation: Transfemoral versus Transapical Approach. Ann. Thorac. Surg..

[19] Siregar S., de Heer F., Groenwold R.H.H., Versteegh M.I.M., Bekkers J.A., Brinkman E.S., Bots M.L., van der Graaf Y., van Herwerden L.A. (2014). Trends and Outcomes of Valve Surgery: 16-Year Results of Netherlands Cardiac Surgery National Database. Eur. J. Cardio-Thorac. Surg..

[20] Grant S.W., Kendall S., Goodwin A.T., Cooper G., Trivedi U., Page R., Jenkins D.P. (2021). Trends and Outcomes for Cardiac Surgery in the United Kingdom from 2002 to 2016. JTCVS Open.

[21] Brown K.L., Crowe S., Franklin R., McLean A., Cunningham D., Barron D., Tsang V., Pagel C., Utley M. (2015). Trends in 30-Day Mortality Rate and Case Mix for Paediatric Cardiac Surgery in the UK between 2000 and 2010. Open Heart.

